# How Dark Stars and Three Stripes Can Aid in Characterization of Muscle Sarcoidosis?

**DOI:** 10.5334/jbsr.4165

**Published:** 2025-12-22

**Authors:** Rob Vernelen, Charlotte Vanhoenacker, Filip Vanhoenacker

**Affiliations:** 1 UZ Leuven, University of Leuven, Leuven, Belgium; 2 AZ Sint‑ Maarten, Mechelen, Belgium; 3 AZ Sint‑Maarten, Mechelen, Faculty of Medicine and Health Sciences, University of Antwerp and Gent, Belgium

**Keywords:** sarcoidosis, muscle sarcoidosis, MRI, US, dark star, three stripes

## Abstract

*Teaching point:* The clues to the correct diagnosis of muscle sarcoidosis on MRI are the typical ‘dark star’ sign in the axial plane and the ‘three‑stripe’ sign in the longitudinal plane.

## Case History

A 36‑year‑old male presented with swollen calves and multiple palpable nodules in the calf muscles. Medical history revealed sarcoid‑related uveitis and nonspecific enlarged mediastinal lymph nodes.

**Figure 1:**
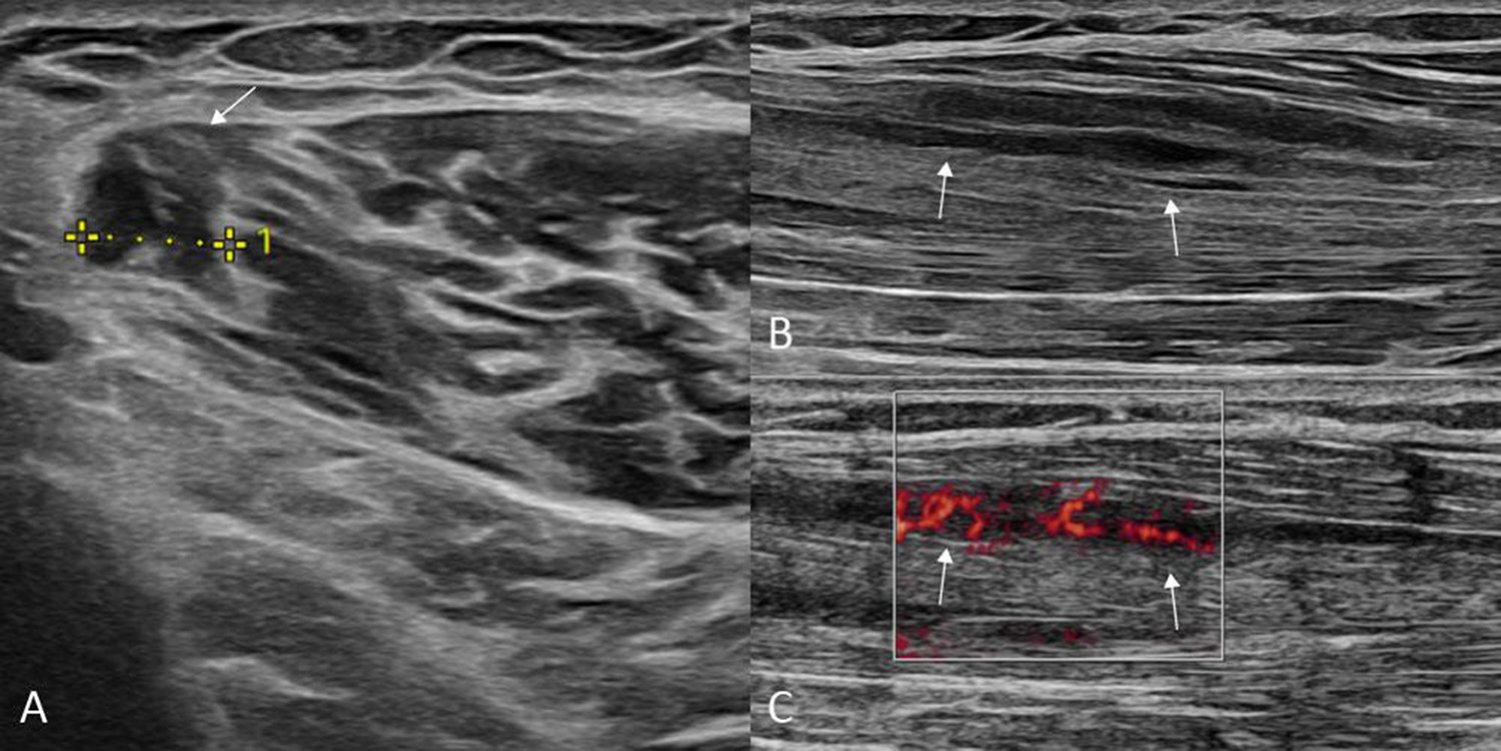
Axial **(A)** and longitudinal **(B)** US images showing an echogenic lesion (arrows) within the calf muscle surrounded by a hypoechogenic periphery. Longitudinal power Doppler **(C)** imaging shows hyperemia suggestive of inflammation.

Ultrasound (US) revealed multiple echogenic lesions within the calf muscles surrounded by a hypoechogenic periphery on axial (Figure 1A, arrow) and longitudinal (Figure 1B, arrows) images, orientated along the muscle axis with increased peripheral flow signal on longitudinal power Doppler imaging (Figure 1C, arrows), most likely indicating inflammation.

**Figure 2:**
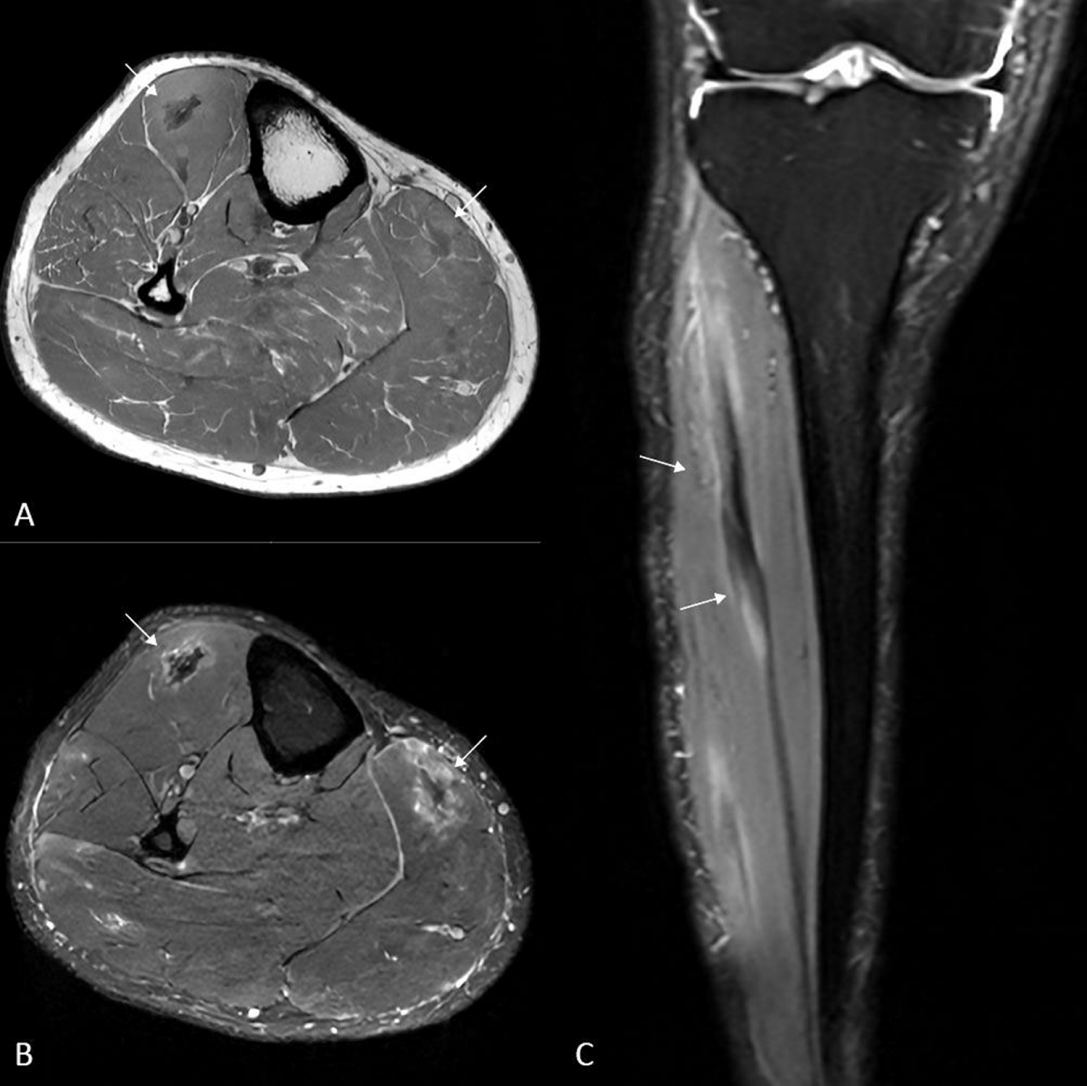
Axial T1‑WI **(A)** and T2‑WI **(B)** showing multifocal intramuscular lesions (arrows) with central hypointense and peripheral hyperintense signals resembling a dark star. The corresponding coronal T2‑WI **(C)** shows the same lesion with the characteristic three‑stripe configuration.

**Figure 3:**
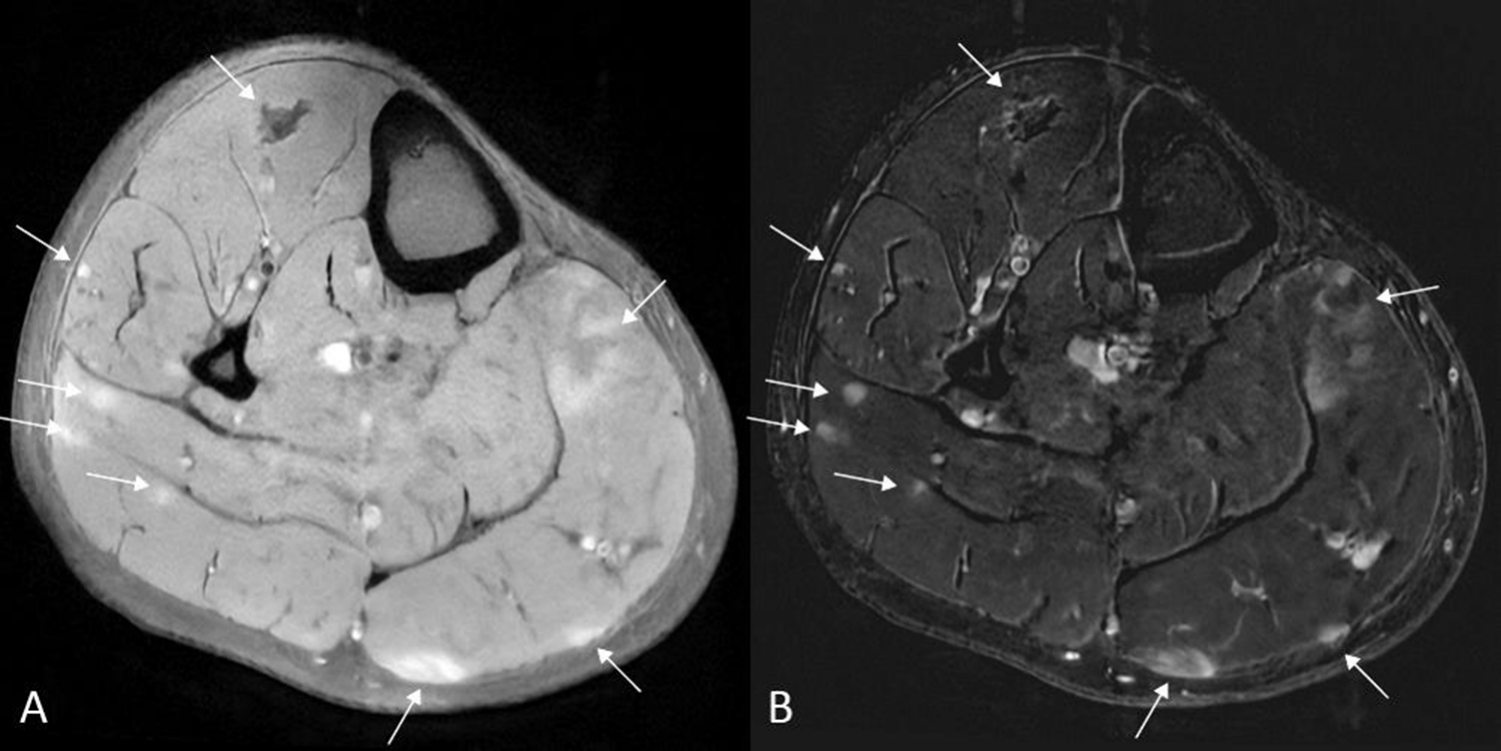
Axial FS T1‑WI **(A)** shows hyperintense lesions (arrows) that become more conspicuous after contrast administration; axial subtraction imaging **(B)** depicts the contrast enhancement.

On magnetic resonance imaging (MRI), multifocal intramuscular lesions were observed with central hypointense and peripheral hyperintense signals on axial T1‑weighted images (WI) (Figure 2A, arrows), more pronounced on T2‑WI, resulting in a ‘dark star’ and ‘three‑stripe’ morphology on axial (Figure 2B, arrows) and coronal (Figure 2C, arrows) images, respectively. The lesions were hyperintense on fat‑suppressed (FS) T1‑WI (Figure 3A, arrows). Subtraction images before and after intravenous gadolinium contrast administration revealed enhancement (Figure 3B, arrows). Central diffusion restriction was seen on diffusion‑weighted images (DWI) (not shown).

## Comments

Sarcoidosis is a multisystem inflammatory disorder characterized by noncaseating granulomas in multiple organs, predominantly the lungs and mediastinal lymph nodes. Muscle involvement is rare and often asymptomatic.

US is the initial imaging modality. US findings include echogenic lesions with a hypoechogenic rim and disturbance of fibrillar muscle architecture due to inflammatory infiltration and fibrotic remodeling. Intramuscular lesions may regress or resolve under corticosteroid therapy.

However, MRI is preferred for soft tissue characterization. On MRI, active inflammation presents as areas of high signal intensity on FS T2‑WI, whereas fibrotic or scar tissue is of low signal on FS T2‑WI [1]. Muscular sarcoidosis may show characteristic MRI patterns—the ‘dark star’ or ‘three‑stripe’ signs—aiding specific diagnosis. [1]. The ‘dark star’ sign on FS T2‑WI represents a centrally hypointense, star‑shaped lesion corresponding to foam cells and fibrotic tissue surrounded by a hyperintense, inflammatory rim. The ‘three‑stripe’ sign is the longitudinal equivalent of a central, hypointense inner stripe on FS T2‑WI, delineated by two adjacent peripheral hyperintense outer stripes on FS‑T2‑WI that enhance after administration of gadolinium contrast.

The differential diagnosis includes polymyositis, muscle strain, delayed onset muscle soreness (DOMS), denervation edema, and diabetic muscle infarct (DMI). Prompt recognition of dark star and three‑stripe signs can enable confident diagnosis and may prevent unnecessary biopsy.
